# Impacts of Environmental Factors on Head and Neck Cancer Pathogenesis and Progression

**DOI:** 10.3390/cells10020389

**Published:** 2021-02-13

**Authors:** Marisol Miranda-Galvis, Reid Loveless, Luiz Paulo Kowalski, Yong Teng

**Affiliations:** 1 Department of Oral Biology and Diagnostic Sciences, Dental College of Georgia, Augusta University, Augusta, GA 30912, USA; mgalvis@augusta.edu (M.M.-G.); rloveless@augusta.edu (R.L.); 2 Department of Head and Neck Surgery and Otorhinolaryngology, A.C. Camargo Cancer Center, São Paulo 01509, Brazil; lp_kowalski@uol.com.br; 3 Head and Neck Surgery Department, Medical School, University of São Paulo, São Paulo 01509, Brazil; 4 Georgia Cancer Center, Department of Biochemistry and Molecular Biology, Medical College of Georgia, Augusta University, Augusta, GA 30912, USA; 5 Department of Medical Laboratory, Imaging and Radiologic Sciences, College of Allied Health, Augusta University, Augusta, GA 30912, USA

**Keywords:** environmental factors, head and neck cancer, metastasis, anticancer, modern therapy

## Abstract

Epidemiological and clinical studies over the past two decades have provided strong evidence that genetic elements interacting with environmental components can individually and collectively influence one’s susceptibility to cancer. In addition to tumorigenic properties, numerous environmental factors, such as nutrition, chemical carcinogens, and tobacco/alcohol consumption, possess pro-invasive and pro-metastatic cancer features. In contrast to traditional cancer treatment, modern therapeutics not only take into account an individual’s genetic makeup but also consider gene–environment interactions. The current review sharpens the focus by elaborating on the impact that environmental factors have on the pathogenesis and progression of head and neck cancer and the underlying molecular mechanisms involved. Recent advances, challenges, and future perspectives in this area of research are also discussed. Inhibiting key environmental drivers of tumor progression should yield survival benefits for patients at any stage of head and neck cancer.

## 1. Introduction

Each year, 450,000 global deaths are attributed to head and neck cancers [1]. Of these, head and neck squamous cell carcinoma (HNSCC), arising from mucosal surfaces of the oral cavity, oropharynx, larynx, and hypopharynx, represents the most common histological subtype [2]. Although these tumors originate from the same squamous epithelium, HNSCC is nevertheless a biologically and clinically heterogeneous disease involving different risk factors, molecular pathogeneses, treatment responses, and prognoses [3,4].

The alarming rates of mortality reported in HNSCC are, at least in part, due to the high prevalence of loco-regional recurrence and/or metastatic disease [5]. Indeed, while patients affected with locally advanced HNSCC present 5-year overall survival rates under 50%, subjects with early stages of the disease carry a markedly improved prognosis, with survival rates closer to 80% [6]. Ever since initial descriptions of HNSCC metastasis were reported in the early 19th century, researchers have abandoned the concept of passive drainage of tumor cells into regional lymph nodes [7]. Instead, metastasis has been recognized as a complex, multi-step process that is orchestrated by tumor biology and supported by the internal tumor microenvironment, as well as external environmental factors that are involved in tumor cell invasion, intravasation, circulation, extravasation, and metastatic colonization [8].

**Figure 1:**
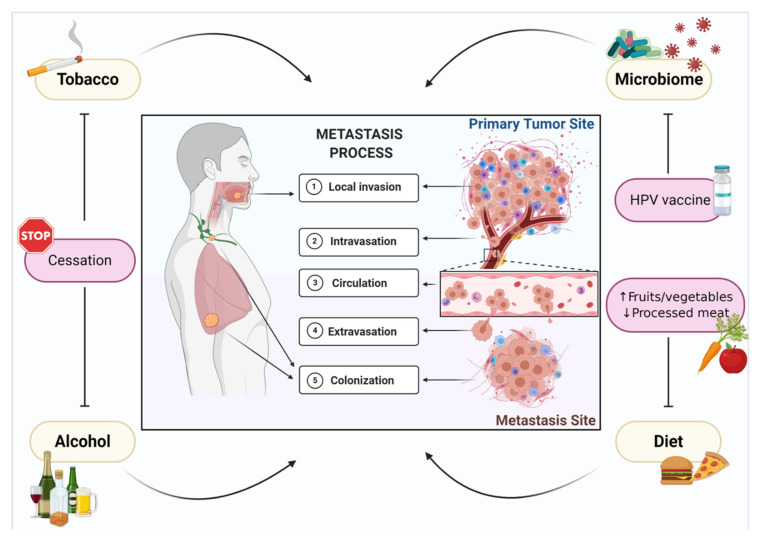
Environmental factors support metastasis of head and neck cancer. Metastasis is a complex and multi-step process that is orchestrated by tumor biology and supported by external environmental factors, such as tobacco and alcohol consumption, human papillomavirus (HPV) infection, the microbiome, and diet, which are involved in tumor cell invasion, intravasation, circulation, extravasation, and metastatic colonization. A precision-based model of metastasis prevention centered around modifiable risk factors involves smoking and alcohol cessation, HPV vaccination, and a diet that is high in fruits and vegetables and low in processed and red meats.

While the role of the tumor microenvironment has been the subject of intensive research in recent years, far less is known regarding the participation of external factors in metastasis. Besides the two greatest risk factors, tobacco and alcohol consumption, oncogenic viruses (e.g., human papillomavirus (HPV)), the microbiome, and diet have also been established in recent decades as contributing factors (Figure 1). The current review provides a comprehensive summary of the environmental factors involved in HNSCC and highlights the recently reported mechanisms involved in environmental factor-associated HNSCC progression. Evidence supporting the further development of a precision-based model of cancer prevention hinged on modifiable risk factors is also provided.

## 2. Tobacco Smoking

A sudden spike in deaths related to lung carcinoma in 1950 led R. Doll and B. Hill to identify an increased risk of cancer among tobacco-smoking patients [9]. Afterward, data from several studies extended the association of tobacco consumption with carcinomas affecting the head and neck, esophagus, pancreas, bladder, kidney, cervix, and stomach, as well as with cardiovascular and respiratory disease.

Cigarettes, the most common form of tobacco, contain over 7000 chemicals and toxic substances, including more than 60 recognized carcinogens [10]. Direct evidence based on experimental animal models shows *N*′-nitrosonornicotine (NNN), a tobacco-specific nitrosamine (TSNA), to be the major tobacco component driving head and neck carcinogenesis [11]. Alongside NNN, other TSNAs and tobacco constituents, such as polycyclic aromatic hydrocarbons (PAHs), aromatic amines, and certain volatile organic agents, contribute to tumorigenesis and tumor development [10]. Underlying tobacco’s pro-tumor contributions is the creation of an imbalance between the metabolic activation and detoxification of carcinogens that directly leads to DNA damage. Metabolic activation is primarily achieved through cytochrome P450 enzymes (CYPs), while metabolic detoxification can be performed by a range of enzymes, like glutathione-S-transferases (GSTs) and uridine-5′-diphosphate-glucuronosyltransferases (UGTs) [12]. DNA adducts produce specific DNA mutations that, if left unrepaired, can activate oncogenes and/or inactivate tumor suppressor genes. In almost all HNSCC smoking-related tumors, integrated genomic annotation of molecular alterations shows a loss of function of the tumor suppressor gene p53 and the inactivation of the cyclin-dependent kinase inhibitor 2A (CDKN2A) [3].

A large body of evidence has pointed to tobacco consumption as the major environmental risk factor for the development of HNSCC. Indeed, patients who report tobacco consumption are 2.13 times more likely to develop HNSCC in comparison with those who have never used tobacco [13]. Moreover, the risk of cancer-related deaths in patients affected by HNSCC is 36% higher in smokers than in non-smokers [14], which supports the findings that smokers present a significantly lower overall survival [15]. Despite smoking cessation showing considerable advantages within the first 4 years, a time frame closer to 20 years is considered to be required for a patient to carry the same risk level as a patient who has never smoked [16]. Taken together, the long-term and highly damaging effects of tobacco carcinogens on the mucosal epithelia have been made clear.

Over time, research has revealed the important roles of tobacco components throughout the metastatic process. This is reflected by the fact that patients who continue smoking during cancer treatment exhibit higher rates of distant metastasis than former smokers or never smokers (31% versus 4%, respectively) [17]. This association also expands into patients presenting HPV-related tumors, a subset that will be discussed in the next section. Although HPV-associated HNSCC is characterized by improved outcomes, smokers presenting HPV positive tumors have a five times higher chance of developing distant metastases when compared with non-smokers with HPV-related tumors [18].

**Figure 2:**
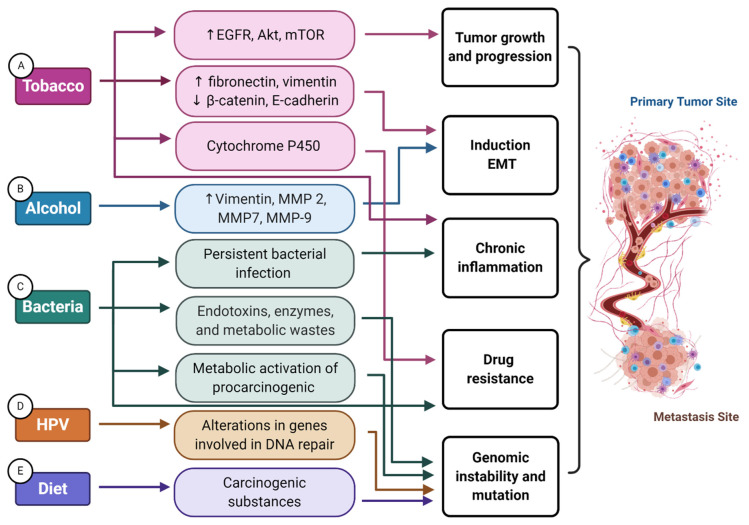
Mechanisms involved in environmental factor support of the onset and progression of head and neck cancer. (**A**) The expression of alpha-7 nicotinic acetylcholine receptors (nAChRs) promotes proliferation and migration through the phosphorylation of epidermal growth factor receptor (EGFR), protein kinase B (Akt), mammalian target of rapamycin (mTOR), and the stimulation of beta-adrenergic receptors. Nicotine upregulates the expression of mesenchymal proteins (fibronectin and vimentin), whereas it downregulates epithelial proteins (beta-catenin and E-cadherin), thereby supporting cell motility and invasion through induction of epithelial–mesenchymal transition (EMT). Nicotine can perturb drug efficacy via cytochrome P450 (CYP)-mediated metabolism, glucuronidation, and/or protein binding, which may impact the efficacy of anticancer drugs. Tobacco consumption also promotes a pro-inflammatory tumor microenvironment, further supporting tumor growth. (**B**) Alcohol is capable of directly upregulating vimentin, matrix metalloproteinase (MMP)-2, MMP-7, and MMP-9, promoting an EMT invasive phenotype and extracellular matrix remodeling. (**C**) Particular bacteria participate in the metabolic activation of carcinogenic chemicals, like acetaldehyde, that can promote tumorigenesis through genomic mutations. Chronic inflammation prompted by persistent bacterial infection also supports multiple hallmark capabilities. Bacteria products like endotoxins, enzymes, and metabolic wastes might cause DNA damage, consequently altering cell cycle control and signaling pathways that can lead to even further genomic instability and mutation. Certain immune cell responses to gut commensal bacteria are also associated with immunotherapy response. (**D**) HPV-related tumors carry more frequent alterations in genes involved in DNA repair, such as PRKDC, potentially hindering a cell’s capacity for DNA repair. (**E**) Lastly, red meat and processed meats also contain carcinogenic substances that can cause genomic instability and mutations.

Tobacco smoking may contribute to cancer progression and metastasis in different ways, such as by inducting an epithelial–mesenchymal transition (EMT)-like phenotype, promoting a pro-inflammatory tumor microenvironment [12], or altering or blocking the pharmacokinetics of anticancer drugs (Figure 2A) [19]. The expression of alpha-7 nicotinic acetylcholine receptors (nAChRs) on tumor cells promotes proliferation and migration through the phosphorylation of epidermal growth factor receptor (EGFR), protein kinase B (Akt), mammalian target of rapamycin (mTOR), and the stimulation of beta-adrenergic receptors [19,20,21]. Nicotine upregulates the expression of mesenchymal marker proteins, like fibronectin and vimentin, but downregulates the epithelial marker proteins beta-catenin and *E*-cadherin [22]. In addition, nicotine can perturb drug efficacy via CYP-mediated metabolism, glucuronidation, and/or protein binding. Emerging data further shows that nicotine exposure contributes to the development of metastasis by supporting the mechanisms driving perineural invasion (PNI) [23]. PNI is a recently recognized pathway involved in the spread of solid tumors and associated with a substantially high risk of local recurrences, metastasis, and decreased survival [24]. Although the underlying mechanisms implicating PNI are not entirely understood, emerging evidence based on the analysis of human cancer biopsies and experimental animal models has revealed that this complex process, called neural tracking, is driven by molecular signaling between neuronal and tumor cells via neurotrophic factors [24]. Overexpression of the nerve growth factor (NGF) and its receptor tropomyosin-related kinase A (TrkA) was reported in HNSCC patients affected with PNI-positive tumors compared to their PNI- negative counterparts [25]. In this perspective, activation of the brain-derived neurotrophic factor (BDNF)/TrkB axis in HNSCC supports cell proliferation, invasion, and EMT [26]. Interestingly, Trk-targeted therapy decreases tumor cell growth and migration, as well as sensitizes them to cisplatin therapy [27]. A previous study has shown that tobacco consumption can stimulate neurotrophic factors and their receptors in a dose-dependent manner [23]. Therefore, HNSCC patients who are current or ex-smokers present a higher prevalence of PNI than HNSCC patients who have never smoked.

## 3. Alcohol

At first glance, alcohol produces epithelial atrophy and decomposes cell lipid components, facilitating the absorption of carcinogens obtained from tobacco (primarily NNN), diet, or other sources into epithelial cells [28]. The major metabolite of ethanol metabolism, acetaldehyde, is shown to be highly mutagenic. Nevertheless, the role of alcohol consumption as an independent factor in HNSCC development is not corroborated in the current literature. Otherwise, the synergistic consumption of tobacco and alcohol has been widely recognized as the main risk factor for HNSCC. Therefore, it appears that alcohol acts as a tumor progression promoter rather than a carcinogen [29,30].

The expression of hypoxia-inducible factor 1-alpha (HIF-1α), a protein central to controlling hypoxic tumor microenvironments, has been reported to be higher in tumor specimens obtained from patients affected by oral cancer who endorse alcohol consumption than those who deny it [31]. Importantly, HIF-1α upregulates the transcription of a wide number of factors involved in promoting invasive and metastatic properties in tumor cells, like EMT and angiogenesis. Data from colon and breast cancer research revealed that alcohol is capable of directly upregulating vimentin, matrix metalloproteinase (MMP)-2, MMP-7, and MMP-9, promoting the EMT invasive phenotype through the EGFR-Snail-mediated pathway [32] (Figure 2B). Furthermore, in vitro studies evaluating lung, colon, and breast cancer cells unveiled that ethanol induces tumor hematogenic dissemination through the formation of actin stress fibers and disrupts junctional vascular endothelial (VE)–cadherin integrity, enhancing tumor cell invasion through blood vessel disruption during the metastatic process [33].

## 4. Microbiome

The oral microbiome is a diverse arena composed of approximately 1000 different microbes, including bacteria and viruses, which exist in a functional equilibrium with the host under normal conditions. Nevertheless, certain conditions prompt the disruption of this equilibrium, leading to the development of several systemic and local disorders such as malignant tumors [34].

### 4.1. Viruses

The first evidence of viruses promoting tumorigenesis dates back to 1964 when the Epstein–Barr virus (EBV) was found to be associated with Burkitt lymphoma [35]. Nowadays, seven oncogenic viruses have been recognized: EBV (further related to Hodgkin lymphoma, Burkitt’s lymphoma, gastric cancer, and nasopharyngeal carcinoma), hepatitis B virus (HBV, related to hepatocellular carcinoma), hepatitis C virus (HCV, related to hepatocellular carcinoma), human immunodeficiency virus (HIV, related to Kaposi sarcoma, non-Hodgkin and Hodgkin lymphomas), human herpesvirus 8 (HHV-8, related to Kaposi sarcoma), HPV (related to cervical, vaginal, vulvar, penile, anal oropharynx carcinomas, and bladder cancer), and human T-lymphotropic virus (HTLV-1, related to adult T cell leukemia/lymphoma) [36].

#### 4.1.1. EBV

EBV (also called *Human gammaherpesvirus 4*) has been associated with various human malignancies, including nasopharyngeal carcinoma (NPC) [36]. NPC is an unusual tumor that arises from the nasopharyngeal epithelium and most commonly affects the nasopharynx [2]. NPC occurs most often as an advanced disease with high locoregional infiltration and lymphatic and distant metastasis. Approximately 30% of cases relapse after treatment. The high degree of aggressiveness may be explained, in part, due to the poorly differentiated or undifferentiated histological features, besides the abundant lymphatic network found in the nasopharynx that allows early lymphatic invasion [37]. The most common sites of distant metastases, occurring in around 5% of NPC patients, are the bones, followed by lung, liver, and distant lymph nodes [2].

EBV infection exerts a key function in tumor onset and progression through the regulation of multiple processes, including modifying epigenetic profiles, inducing genomic instability, evading immune response, promoting cell survival, and contributing to stem-cell-like properties [38]. LMP1, the major oncoprotein encoded by EBV, is one of the key latency II gene products that are related to every critical aspect of tumor biology, mainly through nuclear factor kappa B (NF-κB) activation [39]. Promotion of metastasis in NPC is orchestrated by LMP1, which regulates a cascade of molecular signaling involving MMP-9, mucin 1 (MUC1), vascular endothelial growth factor (VEGF), cyclooxygenase 2 (COX-2), fibroblast growth factor 2 (FGF-2), and HIF-1α [39]. Besides LMP1, novel genes such as TP53, RAS, and microRNAs encoded by EBV (i.e., BART2-5p) have been recently identified to have important roles in NPC metastasis [40].

While EBV infection was established as the driving factor for non-keratinizing NPC (NK-NPC) as early as 1973, its counterpart, keratinizing NPC (K-NPC), lacks association with EBV, mostly in non-endemic regions. Instead, tobacco smoking and alcohol consumption are recognized as the main causative carcinogens for this histologic type. Other risk factors for NPC include host genetic susceptibility and exposure to nitrosamines from salted and fermented foods, particularly from consumption in early life [37].

#### 4.1.2. HPV

The molecular mechanisms of HPV-related HNSCC carcinogenesis involve the insertion of genomic HPV DNA into basal epithelial cells, leading to the expression of the viral oncoproteins E6 and E7. Consequently, key cellular signaling pathways responsible for cell cycle control are altered through the degradation of the tumor suppressor protein p53 via E6 and retinoblastoma protein (pRb) via E7, resulting in cell malignant transformation and immortalization [41]. Furthermore, HPV E6 protein interacts with c-myc constituting the complex c-myc/E6, which activates the transcription of the human telomerase catalytic subunit of (hTERT), contributing to tumor cell immortalization [42].

High-risk HPV (subtypes 16, 18, 33, and 52) infection is well established as an etiological factor for HNSCC. HPV-related HNSCC presents unique molecular, clinical, and pathologic features compared to tobacco-related tumors. While the overall incidence rates of HNSCC associated with tobacco and alcohol consumption have declined over recent years, patients with HPV-positive (+) disease are responsible for the increasing prevalence reported in some countries [43]. Encouragingly, preventive approaches, such as prophylactic HPV vaccination, can decline the prevalence of HPV infection by 88.2% [44]. In particular, HPV (+) disease predominantly affects patients younger than 45 years old, the tumors typically involve the oropharynx and present advanced lymph node metastasis, and the patients generally show improved prognosis. Histopathologic analysis reveals that non-keratinized tumors exhibit a basaloid morphology, while molecular profiling indicates a lack of mutations in the TP53 gene. Based on this, the American Joint Committee on Cancer (AJCC) pathologic staging system separated HPV (+) and HPV-negative (−) HNSCC tumors into two different subgroups in the last edition released [45,46].

Apart from its function as an etiologic factor, the clinical relevance of HPV infection has been made evident through well-conducted clinical trials showing HPV status as an independent prognostic factor for oropharynx squamous cell carcinoma [47]. In particular, the incidence rates of second primary tumors and distant metastasis among patients with HPV (+) tumors were seen to be significantly lower than those with HPV (−) tumors. Accordingly, patients with HPV-related tumors showed improved overall survival (82.4% at 3 years) and a 58% reduction in the risk of death compared to subjects with HPV (−) tumors (57.1% at 3 years) [47]. The clinical relevance also implicates new strategies in de-escalation treatment for patients with HPV (+) tumors to reduce treatment-related toxicities, morbidity, and costs [48].

Recent research has led to elucidating the various biological and clinical mechanisms implicated in the higher survival rates among patients with HPV-related tumors. (1) Although HPV (+) and HPV (−) tumors share similar molecular pathways driving tumorigenesis, HPV-related HNSCC presents a lower genetic mutational profile [3]. (2) Patients affected with HPV (+) tumors also have a higher sensitivity to radiation therapy, resulting in superior local and regional control [49]. (3) Moreover, HPV (+) tumors contrast tobacco- and alcohol-associated HNSCC, which develop more frequent synchronous primary tumors and second primary tumors at different sites covered by squamous epithelium (e.g., oral cavity, pharynx, larynx, esophagus, and lungs) due to molecular alterations and long-term exposure to environmental carcinogens (a concept known as field cancerization) [50]. (4) From a microenvironmental perspective, the improved clinical response of HPV-related tumors may further be explained by the adaptive immune responses against viral antigens that stimulate potent antitumor immunity [51]. Therefore, patients affected by HPV (+) HNSCC present an improved response to immunotherapy [52]. (5) Lastly, the clinical profile of patients with HPV (+) tumors, such as younger subjects with fewer comorbidities, favors oncologic management.

As mentioned previously, tobacco consumption has the potential to modify the biological and clinical behavior of HPV-related tumors. Thus, patients that present with both HPV (+) tumors and more than 10 pack-years of tobacco smoking are classified in the intermediate stage of risk of death, similar to those patients with HPV (–) tumors at the early tumor stages with less than 10 pack-years of tobacco smoking [47].

Extensive research has established that HPV-related HNSCC presents an improved overall survival compared to its HPV (−) counterpart. Despite the fact that some studies have reported similar metastatic patterns between patients with HPV (+) and HPV (−) tumors [47,53,54], others have recognized considerable differences. In comparison with patients with HPV (−) tumors, the rate of distant metastases in HPV (+) tumor patients is lower (11.1% versus 23.1%), and the development time of distant metastases is longer (median 16.4 versus 7.2 months). Distant metastases arising from HPV (+) tumors tend to involve more sites (2.04 versus 1.33 sites), and the anatomical locations involved (brain, kidney, skin, skeletal muscle, and non-regional lymph nodes) are atypical for head and neck cancer [55,56,57]. The underlying mechanisms involved in the unusual pattern of distant metastases are still under investigation. Data coming from genomic characterization suggest that metastatic HPV-related tumors carry more frequent alterations in genes involved in DNA repair, such as the protein kinase, DNA-activated, catalytic subunit (PRKDC) (Figure 2D). Phosphoinositide 3-kinase (PI3K) pathway alterations are also associated with improved survival [58]. Moreover, in vitro and in vivo HNSCC xenograft models combined with patient data have revealed two different ways by which p16, the surrogate marker for HPV infection, contributes to different dissemination patterns: (1) regulating vascular invasiveness and angiogenesis and (2) stimulating nodal spread by increasing lymphatic vessel formation via alpha4 beta1 integrin upregulation [59].

### 4.2. Bacteria

The association between bacterial infection and cancer development was initially identified between *Helicobacter pylori* (*H. pylori*) and gastric cancer. Nowadays, the World Health Organization/International Agency for Research on Cancer (IARC) has recognized *H. pylori* as a definite carcinogen for humans. Subsequently, *H. pylori* infection has also been linked to low-grade gastric-mucosa-associated lymphoid tissue (MALT) lymphoma, *Salmonella typhi* infection with gallbladder cancer, *Chlamydia trachomatis* with cervical cancer, and *Chlamydia pneumoniae* with lymphoma and lung cancer [60]. The underlying mechanisms involved include a strong host immune response triggered by the bacterial infection, causing chronic inflammation, metaplasia, dysplasia, and, lastly, malignant transformation [61]. Specifically, *H. pylori* generate chromosomal translocations in infected cells [62], and the carcinogenic toxins produced by *Salmonella typhi* deregulate cell cycle control and DNA repair mechanisms [63], contributing to the carcinogenic process.

Despite the oral cavity constituting one of the most diverse and complex microbiomes, the role of dysbiosis in HNSCC development and progression has only recently been placed under investigation [64]. Initial studies have identified the prevalence of Gram-negative anaerobes to be twice as high in patients affected with oral squamous cell carcinoma (OSCC), alongside a decrease in bacterial abundance, diversity, and taxonomic composition compared to healthy subjects [65,66]. Further characterization of the OSCC microbiome reveals a microbial profile enriched by opportunistic pathogens, including *Fusobacterium nucleatum*, *Prevotella intermedia*, *Aggregatibacter segnis*, *Peptostreptococcus stomatis*, and *Catonella morbi* [67]. Most surprisingly, and for the first time, a microbial signature involving the high prevalence of *Lactobacillus* and/or the low incidence of *Haemophilus, Neisseria, Gemellaceae,* or *Aggregatibacter* in saliva has been suggested as a biomarker for HNSCC.

Given the wide effects of tobacco smoking in the oral cavity, it is not surprising that tobacco consumption also leads to direct alterations in the oral microbial composition. Indeed, smoker subjects, independent of alcohol ingestion, show lower species richness, including a decrease in the abundance of *Neisseria, Gemella*, and *Peptostreptococcus* [34]. Besides the impact of tobacco on the oral cavity microflora, HPV infection is also considered a major etiologic risk factor for oropharyngeal cancer and to drive changes in bacterial ecology. In particular, the oral microbiome profile in HPV-related oral cancer is represented by a richness of *Lactobacillus* and *Weeksellaceae* [65]. Of note, species adapted to hypoxic conditions distinctive of the tumor microenvironment, such as *Veillonella, Megasphaera*, and *Anaerolineae,* have been recognized as potential biomarkers for HPV-related HNSCC [65,68].

Currently, the literature to date fails to provide a cause–effect association between the oral microbiome and the carcinogenesis of HNSCC. Nevertheless, emerging evidence suggests that the altered microflora identified in HNSCC patients may play an important role in shaping the tumor microenvironment through different mechanisms [69]. First, particular bacteria participate in the metabolic activation of pro-carcinogenic chemicals, like acetaldehyde (a major ethanol metabolite), that are indispensable to host cell–molecule interactions [69]. Moreover, chronic inflammation prompted by persistent bacterial infection supports multiple hallmark capabilities of carcinogenesis [70]. In particular, bacterial products like endotoxins, enzymes, and metabolic wastes might also cause DNA damage, consequently altering cell cycle control and signaling pathways that induce mutations of tumor suppressor genes and promote the activity of proto-oncogenes [71].

Recently, research using 16S ribosomal DNA (rDNA) amplicon sequencing has highlighted the alterations in the microbiome composition implicated in HNSCC progression [65,72]. The enriched presence of *Lactobacillus, Actinomyces* and *Parvimonas* was associated with HNSCC in advanced clinical stage (TNM) and advanced tumor stage, respectively [65,72]. Conversely, novel findings based on animal models and human patients revealed that certain immune cell responses to gut commensal bacteria, specifically, *Bacteroides thetaiotaomicron* and *Bacteroides fragilis,* are associated with an anti-cytotoxic T-lymphocyte-associated protein 4 (CTLA-4) immunotherapy response [73]. Concordant with this notion, oncology management with drugs targeting the programmed death-ligand 1 (PD-L1) pathway enhanced with the oral administration of *Bifidobacterium* was found to significantly improve the efficacy of immunotherapy [74] (Figure 2C).

Although the etiologic role of the oral microbiota in HNSCC development and progression is still under investigation, the available data indicate that the identification, characterization, and further manipulation of microbiome components may contribute to the control of malignant progression and metastases.

## 5. Diet and Nutrition

The term diet refers to the type and total amount of food and drinks regularly consumed by an organism. Diet provides the nutrients required for the biochemical reactions involved in metabolic processes aimed at producing the energy needed for cellular functions. It is imperative to recognize, however, that some non-nutrient substances (e.g., chemicals, caffeine) obtained through a diet are also capable of impacting cell metabolism. The set of stages implicated in the biological processes involving ingestion, digestion, absorption, transport, assimilation, and excretion are denominated nutrition [75].

A compelling body of research has recognized the critical role of diet in several chronic diseases and conditions, including obesity, diabetes, cardiovascular diseases, osteoporosis, and dental and periodontal diseases [76]. Historically, data regarding the influence of diet and nutrition on cancer risk has been uncertain, at least in part, due to the multiple exposures involved, the wide anatomical sites that can be affected, and the long time frame between the cause (exposure) and the effect (tumor development) [77]. Nevertheless, the Third Expert Report of the World Cancer Research Fund (WCRF) and the American Institute for Cancer Research (AICR) has recently compiled strong evidence indicating an important relationship between diet and nutrition and the development and progression of cancer affecting the head and neck, among others, such as the stomach, lung, liver, kidney, breast, and prostate [78].

### 5.1. Vegetables and Fruits

A wide number of studies developed in different countries across the world with diverse diets, such as Brazil [79], China [80], Italy [81], the Netherlands [82], Taiwan [83], the United Kingdom [84], and the United States [81,85], have provided robust data suggesting an inverse association between the consumption of vegetables and fruits and the risk of HNSCC. In particular, patients reporting a diet without vegetables and fruits had double the probability of developing HNSCC when compared with subjects reporting daily intake of them [83]. Similarly, research evaluating more than 35,000 individuals found lower cancer risks to be correlated with higher vegetable and/or fruit intake [86].

Fruits and vegetables are composed of several bioactive compounds categorized into phytochemicals (e.g., phenolics, flavonoids, carotenoids), micronutrients (vitamins and minerals), and fiber [78]. Most of these components can influence different stages of cancer onset and progression. One family of compounds abundant in plants, polyphenols, has been extensively explored due to their broad functions as antioxidants, anti-inflammatories, and immune regulators [87]. In particular, antioxidant activity reduces reactive oxygen species (ROS), protects against oxidative stress, favors DNA repair, and stimulates the transcription of genes encoding antioxidant enzymes. Remarkably, the regulation of inflammatory mediators like cytokines and chemokines can promote low-grade chronic inflammation [88].

Numerous cell signaling pathways related to glucose metabolism, gene expression, growth factor transcription, cell cycle intermediates, microRNAs, and epigenetic modifications may be impacted at multiple levels by these phytochemicals (e.g., NF-κB, Akt, mitogen-activated protein kinase (MAPK), Wnt, Notch) [82,87]. In this perspective, in vitro studies evaluating breast cancer cells in response to polyphenols have reported cell cycle arrest during the G1/S and G2/M phases [89]. Indeed, individuals with the greatest intake of carotenoid had a 39% lower risk of developing HNSCC than subjects with low carotenoid consumption [90]. Furthermore, a recent evaluation of different risks among HNSCC subsites suggests an additional local effect produced by the direct contact of food with the squamous cell epithelium, with the strongest association reported in tumors located in the oral cavity [82].

### 5.2. Red Meat and Processed Meat

Although meat is an important source of protein, micronutrients (e.g., vitamin B6, vitamin B12), and minerals (e.g., zinc, iron, selenium, phosphorus), the process of cooking at high temperatures gives rise to the formation of carcinogenic substances, such as PAHs, N-nitroso-compounds (NOC), and heterocyclic aromatic amines (HAA) [78]. In 2015, based on the analysis of more than 800 epidemiological studies, the IARC reported a positive association between the high consumption of red and processed meat and cancer. Specifically, red meat was classified as potentially carcinogenic to humans with strong evidence and was associated with cancer affecting the colon, rectum, pancreas, and prostate. Processed meat, on the other hand, was classified as a carcinogenic agent with sufficient evidence to produce colorectal cancer in humans and was further related to stomach cancer [91] (Figure 2E).

Still, data regarding the relationship between the high consumption of red meat and processed meat and the risk of HNSCC are limited to a few studies with controversial results. A prospective study with a follow-up of over 20 years, however, reported a direct association between high intake of processed meat and HNSCC [92]. Subjects reporting intake of processed meat three or more times a week were found to have a significantly increased risk of HNSCC compared with those without processed foods in their diets [80]. Interestingly, in the subgroup analyzed, a positive association was only seen for tumors located in the oral cavity [92]. In contrast, in a combined analysis including fried foods, processed meats, and sweets, a positive association was only seen for laryngeal cancer [85]. Results from the same study further revealed that a diet including lean protein, fruits, and vegetables overall decreased the risk of HNSCC. To date, however, no studies have found an association between the consumption of red meat and an increased risk of HNSCC [92,93].

## 6. The Influence of Environmental Factors during Cancer Treatment

Environmental factors have not only been found to impact tumor induction and dissemination but patient therapeutic responses as well. Cigarette smoking during treatment, for example, has been associated with increased symptom burden [94] and may cause variable pharmacokinetic perturbations in anticancer agents [95], such as through the transcriptional and epigenetic regulation of metabolic enzymes [96]. Accordingly, smoking during radiation therapy is linked to decreased response rates and survival in patients with head and neck cancer [97]. Interestingly, microbial interactions have also been reported to influence the efficacy and impact of anticancer therapies. *Lactobacillus brevis* CD2 lozenges (commonly found in milk products), for example, were found to reduce the intensity and prevalence of mucositis in HNSCC patients receiving either chemotherapy or radiotherapy, resulting in an increase in treatment completion [98,99]. Aside, increasing evidence from in vivo model systems, as well as patients, suggests that certain gut microbes can positively impact the outcome of cancer immunotherapy through a variety of different mechanisms, like enhancing immunotherapeutic CpG-oligonucleotides and immune checkpoint blockades [100]. Strikingly, recent evidence indicates that certain parasites, like the canine tapeworm *Echinococcus granulosus* (*E. granulosus*), can also play a protective role against cancer. While its mechanism is still under debate, it has been suggested that *E. granulosus* may indirectly perform its anticancer effect through host immune activation [101]. Taken alongside others, such as *Toxoplasma gondii* and *Trypanosoma cruzi*, it is possible that we may one day use the parasites that have historically haunted us to discover novel agents that can be used to regulate various types of cancer [102].

Preclinical and clinical evidence has also begun to emerge on the impact that certain dietary regimens may have on patients during anticancer treatments [103]. Several studies have identified fasting to have a synergistic effect on both chemotherapies and radiotherapies and to possibly reduce treatment toxicities [103]. While likely not as effective, caloric restriction may also provide benefits to those undergoing anticancer therapies. In mice, greater treatment efficacy of vincristine, for example, was seen when a high-fat diet was switched to a low-fat one [104]. Aside, ketogenic diets have sparked great interest and shown to enhance survival and the effectiveness of antitumor drugs, such as carboplatin, in mice [105]. While dietary restrictions/regimens are still open to question, strong arguments for their feasibility and potential benefits as supportive treatment options for head and neck cancer patients has been outlined by Klement [106]. Nevertheless, the biological mechanisms and interactions underlying these factors’ effects on cancer treatments are far from being elucidated and will require further investigation if they are to be employed for higher therapeutic efficacies.

## 7. Conclusions and Perspectives

Cancer is partially caused by changes to certain genes and, in some cases, can directly result from environmental exposures that lead to DNA damage. The evidence presented in the current review supports the notion that environmental factors, including tobacco smoking, alcohol consumption, the microbiome, and HPV infection, can create an environment that is permissive to the genetic molecular circuitry involved in the pathogenesis and progression of HNSCC. Dietary patterns are also a potential risk factor for HNSCC, and there is growing interest in dissecting the relationships between them using objective biomarkers, for example. How environmental factors and their cellular targets reshape processes on the genomic, proteomic, and metabolomic levels has yet to be fully revealed, however, and thus, there remains much to be understood regarding their contribution to HNSCC. Further investigation into the effects of short- and long-term environmental exposures, as well as into early interventions, will likely allow us to better control HNSCC on the individual level through personalized therapy. Nevertheless, creating a culture of prevention that promotes healthy lifestyles may be the most effective (and the most difficult). While continued educational outreach on topics like HPV vaccination will be pivotal to moving forward, multiple health behavior interventions, such as those described by Spring et al. [107], will almost certainly be required alongside investigative efforts to not only combat HNSCC but all cancers.

## Data Availability

No new data were created or analyzed in this study. Data sharing is not applicable to this article.

## Abbreviations

AICR	American Institute for Cancer Research
Akt BDNF	protein kinase B brain-derived neurotrophic factor
CDKN2	cyclin-dependent kinase inhibitor 2A
COX-2	cyclooxygenase 2
CTLA-4	cytotoxic T-lymphocyte-associated protein 4
CYP	cytochrome P450
DNA	deoxyribonucleic acid
EBV	Epstein–Barr virus
EGFR	epidermal growth factor receptor
EMT	epithelial–mesenchymal transition
FGF-2	fibroblast growth factor 2
GSTs	glutathione-*S*-transferases
HAA	heterocyclic aromatic amines
HBV	hepatitis B virus
HCV	hepatitis C virus
HHV	human herpes virus
HIF-1α	hypoxia-inducible factor 1-alpha
HIV	human immunodeficiency virus
HNSCC	head and neck squamous cell carcinoma
H. pylori	Helicobacter pylori
HPV	human papillomavirus
hTERT	human telomerase catalytic subunit
HTLV-1	human T-lymphotropic virus
IARC	International Agency for Research on Cancer
MALT	mucosa-associated lymphoid tissue
MMP	matrix metalloproteinase
MUC1	mucin 1
mTOR	mammalian target of rapamycin
nAChRs	nicotinic acetylcholine receptors
NF-κB	nuclear factor kappa B
NGF	nerve growth factor
NNN	*N′*-nitrosonornicotine
NOC	*N*-nitroso-compounds
OSCC	oral squamous cell carcinoma
PAHs	polycyclic aromatic hydrocarbons
PD-L1	programmed death-ligand 1
PNI	perineural invasion
pRb	retinoblastoma protein
PRKDC	protein kinase, DNA-activated, catalytic subunit
RNA	ribonucleic acid
ROS	reactive oxygen species
Trk	tropomyosin-related kinase
TSNA	nitrosamines
UGTs	uridine-5′-diphosphate-glucuronosyltransferases
VEGF	vascular endothelial growth factor
WCRF	World Cancer Research Fund
